# Transition metal dichalcogenide-based Janus micromotors for on-the-fly *Salmonella* detection

**DOI:** 10.1007/s00604-022-05298-2

**Published:** 2022-04-15

**Authors:** Marta Pacheco, Beatriz Jurado-Sánchez, Alberto Escarpa

**Affiliations:** 1 Department of Analytical Chemistry, Physical Chemistry and Chemical Engineering, University of Alcala, Alcala de Henares, 28871 Madrid, Spain; 2 Chemical Research Institute “Andrés M. del Río”, University of Alcala, Alcala de Henares E-28871, Madrid, Spain

**Keywords:** Janus, Affinity peptides, Micromotors, Fluorescence, Endotoxins

## Abstract

Janus micromotors encapsulating transition metal dichalcogenides (TMDs) and modified with a rhodamine (RhO)-labeled affinity peptide (RhO-NFMESLPRLGMH) are used here for *Salmonella enterica* endotoxin detection. The *OFF–ON* strategy relies on the specific binding of the peptide with the TMDs to induce fluorescence quenching (*OFF state*); which is next recovered due to selectively binding to the endotoxin (*ON state*). The increase in the fluorescence of the micromotors can be quantified as a function of the concentration of endotoxin in the sample. The developed strategy was applied to the determination of *Salmonella enterica* serovar Typhimurium endotoxin with high sensitivity (limits of detection (LODs) of 2.0 µg/mL using MoS_2_, and 1.2 µg/mL using WS_2_), with quantitative recoveries (ranging from 93.7 ± 4.6 % to 94.3 ± 6.6%) in bacteria cultures in just 5 min. No fluorescence recovery is observed in the presence of endotoxins with a similar structure, illustrating the high selectivity of the protocol, even against endotoxins of *Salmonella enterica* serovar Enteritidis with great similarity in its structure, demonstrating the high bacterial specificity of the developed method. These results revealed the analytical potential of the reported strategy in multiplexed assays using different receptors or in the design of portable detection devices.

**Graphical abstract:**

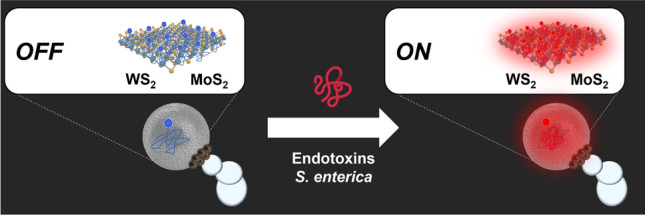

**Supplementary Information:**

The online version contains supplementary material available at 10.1007/s00604-022-05298-2.

## Introduction

Bacterial contamination is a major threat to human health, causing millions of infections worldwide. As such, the development of fast, sensitive, and accurate methods to detect bacteria contamination is of paramount importance. Lipopolysaccharides (LPSs) or endotoxins are the major structural components of the outer membrane of Gram-negative bacteria, which are released during their growth and can be used as biomarkers for bacteria detection [1]. Most LPSs have a structure divided into three chemically differentiable parts covalently bonded together: a polysaccharide moiety called O-antigen, an oligosaccharide core, and finally a lipid region called lipid A. The size of LPSs molecules, therefore, depends on the three sub-units into which it is divided and is usually in the range of 10 and 20 kDa [2]. Indeed, such a unique molecular structure can be used for the on-demand design of specific detection probes in a myriad of methods.

In recent years, there has been a significant interest in the design of new devices for the rapid detection of endotoxins related with main foodborne pathogens, such as *Klebsiella pneumonia*, *Pseudomonas aeruginosa*, *Escherichia coli*, and *Salmonella enterica*, for its application in biodefense, food control, and public health [3]. Among these pathogens, *Salmonella enterica* is the leading cause of foodborne gastroenteritis, causing approximately 94 million infections and 155,000 deaths a year worldwide [4, 5]. Many cases of human salmonellosis are attributed to the consumption of contaminated food with some symptoms such as diarrhea, stomach cramps, nausea, vomiting, and headache, most cases being the result of *Salmonella enterica* serovar Enteritidis or serovar Typhimurium [6, 7]. Currently, the gold standard methods for bacterial contamination detection compromise culture-based methods, polymerase chain reaction (PCR) protocols, which are robust but require expensive and sophisticated instrumentation, qualified personnel, and long analysis times [8]. The *Limulus* amoebocyte lysate (LAL) allows for detection and quantification of endotoxins with excellent LODs ranging from 0.0005 to 5.0 ng/mL, yet such technique suffers from low recoveries and requires blood from the pericardium of horseshoe crabs [9].

Artificial micromotors hold considerable promise for the development of novel sensing strategies for LPS detection, as demonstrated impressive capabilities for the detection of DNA, proteins, pollutants, etc. [10–16]. Micromotors can be propelled by catalytic mechanisms or by external sources, the so-called “fuel-free” micromotors [17]. The motion of catalytic micromotors can be also controlled on-demand with, i.e., ultrasound fields, holding considerable promise for future analytical applications [18]. The self-propulsion capability of such moving particles induces efficient fluid mixing, which is extremely effective in accelerating chemical operations, leading to new *on-the-fly* detection schemes along with higher efficiency, ultrasensitive detection in short times in microscale environments, no sample preparation, and thus potentially lower costs [19]. The potential of fuel-free ultrasound propelled micromotors have been also tested for in vivo biosensing applications [20] and for controlled aggregation in Raman biosensing of nucleic acids [21].

Inspired by the advantages of micromotors in analytical chemistry, our research group has designed polymeric micromotors for the detection of bacterial endotoxins using different strategies. On a first approach, an *ON–OFF* fluorescence detection strategy based on encapsulation of aminophenylboronic acid functionalized graphene quantum dots in the micromotors was used. Endotoxins can interact with quantum dots forming a cyclic ester that causes fluorescence quenching in the micromotor in a concentration dependent manner. The autonomous movement of the micromotor improves the mixing of fluids and the reaction kinetics, enabling the detection of the analyte in less than 15 min, even in viscous samples and media where stirring is not possible. This approach was successfully applied to the detection of *Escherichia coli* endotoxin in clinical samples, such as human serum and urine [22], and *Salmonella enterica* endotoxins in milk, egg, and mayonnaise [23]. The main disadvantage of this strategy is the low selectivity and specificity, recognizing the oligosaccharide core, a fragment in endotoxin common in most Gram-negative bacteria. For this reason, in our research group, two *OFF–ON* micromotor-based biosensing strategies were subsequently developed using transition metal dichalcogenides (TMDs) for the detection of low levels of endotoxins from *Escherichia coli* in connection with fluorescence-labeled affinity peptides, as receptors. The first strategy consists of using tubular micromotors prepared by templated-assisted electrochemical deposition at room temperature of WS_2_ or MoS_2_ in the outer layer using tungstic acid and sulfate or ammonium tetrathiomolybdate exclusively as sources for the synthesis of micromotors, followed attachment of the affinity peptide [24]. The second strategy consists of Janus micromotors synthesized by emulsion self-assembly techniques where exfoliated TMDs were encapsulated in polycaprolactone polymeric bodies for subsequent attachment of the peptide as well [25]. These proposals resulted in highly selective detection strategies with high sensitivity in applications in clinical settings.

Inspired by these results and the potential of affinity peptide modified Janus micromotors for selective endotoxin detection, the aim of this work is to explore the combination of *Salmonella enterica* serovar Typhimurium specific affinity peptide detection probes — designed by phage display — [26] with the Janus micromotors for foodborne pathogens monitoring. To this end, WS_2_ and MoS_2_ have been encapsulated in polycaprolactone (PCL) micromotors, which also contain an asymmetric patch of Pt and Fe_2_O_3_ nanoparticles for efficient propulsion and to facilitate magnetic control. The affinity peptide attaches specifically via a hydrophobic-electrostatic interaction with the MoS_2_ or WS_2_ encapsulated in the micromotors (*OFF state*). Upon the presence of the specific *Salmonella enterica* serovar Typhimurium endotoxin, the affinity peptide detaches from TMD nanomaterial, resulting in an increase in the fluorescence of the micromotor (*ON state*) after just 5-min navigation in microliter-volume samples without any sample treatment. A judicious comparison of the performance of the TMD material used for the encapsulation in terms of analytical characteristics was also performed, revealing the superiority of WS_2_ over MoS_2._ No fluorescence recovery is observed in the presence of *Salmonella enterica* serovar Enteriditis endotoxin or other bacterial endotoxins, illustrating the high selectivity of the protocol. The new strategy allows for fast detection of bacterial contamination and even more, for discrimination among the same family of endotoxins, holding thus considerable promise for multiplexed analysis in connection with affinity peptides labeled with different tags.

### Materials and methods

#### Chemicals

PCL (cat. 440752), iron oxide nanoparticles (20 nm, cat. 700304), chloroform (cat. 650498), poly(ethylene glycol) (PEG, cat. 89510), hydrogen peroxide (30% solution, cat. 216763), tungsten (IV) sulfide (cat. 790583), molybdenum (IV) sulfide (cat. 234842), LPS from *Salmonella enterica serotype* Typhimurium (cat. L6143) and *Salmonella enterica* serotype Enteritidis (cat. L7770) sodium dodecyl sulfate (SDS) (cat. 71727), *Staphylococcus aureus* NCTC 6571 Lenticule® discs (cat. CRM06571M), *Escherichia coli* Strain B (cat. EC11303), chloroplatinic acid hydrate (cat. 398322), hydrazine (30% in H_2_O, cat. 309,400), sodium hydroxide (cat. S5881), and hydrochloric acid (cat. 320331) were purchased from Merck (Germany). The affinity peptides of *Salmonella enterica* (sequence: Rho-NFMESLPRLGMH) were supplied by Quimigen (Madrid, Spain).

#### Instrumentation

Scanning electron microscopy (SEM) and energy-dispersive X-ray mapping analysis (EDX) images were obtained with a JEOL JSM 6335F microscope, with an acceleration of 10 kV and 22 kV, respectively, and a Hitachi T-100 microscope. The fluorescence optical microscopy images and the movement of the micromotors were taken using a Nikon Eclipse Instrument Inc. Ti-S/L100 inverted optical microscope, coupled to a multi-LED light illumination source (CoolLED's pE-4000), a Zyla sCMOS camera, and a G-2A epifluorescence filter (510–560 nm).

#### Affinity peptide-TMD Janus micromotor synthesis

Janus micromotors were synthesized using an emulsion self-assembly technique. In a first step, 4 mL of sodium dodecyl sulfate solution (50 mg) were mixed for 2 h with 5 mg of WS_2_ or MoS_2_ for dispersion using a pulse ultrasound probe with an amplitude of 60% with a program of 2 s on and 1 s off. After this period, they were centrifuged at 13,400 rpm for 15 min. The supernatant was added to a solution of PCL (50 mg), platinum nanoparticles (5 mg, size 50 nm), and iron oxide nanoparticles (20 nm, 150 µL) in 1 mL of chloroform. This mixture was stirred for 10 min and stopped in the open air to evaporate the organic solution overnight. The third and final step is to collect the micromotors and wash them with water for 5 min at 5000 rpm. The resulting micromotors are stable for 3 months. To modify the micromotors with the peptide, 200 µL of micromotors solutions (9000 motors/µL) were incubated with 100 µL of a solution containing 10 µg/mL of affinity peptide under vigorous stirring for 5 min at 25 °C. Next, the solution was washed with ultrapure water by centrifuging twice 5 min at 5000 rpm and finally resuspended in 200 µL of PEG (15%) solution. The modification was performed daily to avoid change in the peptide fluorescence properties.

#### Endotoxin detection

The detection experiment was carried out by mixing 10 µL of the Janus micromotors (final concentration, 3000 micromotors/µL) in 15% PEG solution (final concentration, 5%) with 10 µL of endotoxin solutions (0 to 1000 µg/mL) or bacteria culture and 10 µL of H_2_O_2_ 30% (final concentration, 7.5%) and incubated in a tube for 5 min. After this 5 min, the micromotors are retained with a magnet (to stop the reaction), and the solution is eliminated, replacing it with water to analyze the fluorescence of one drop (of 1 µL approx.) on the microscope. The experiments were carried out in triplicate, analyzing the fluorescence of three independent drops (*n* = 3 drops per assay) taken the average of the individually measured fluorescence in a representative number of micromotors (*n* = 50 motors per drop). Finally, the fluorescence of the assay is the average of the fluorescence obtained in each of the drops. Control experiments without fuel or surfactant were performed similarly.

#### Bacteria culture

*Escherichia Coli* strain B or *Staphylococcus aureus* bacteria were cultivated in Luria–Bertani medium incubated at 100 rpm and 37 °C for 16 h. All experiments followed UAH regulations, and all biosafety measurements were taken.

## Results and discussion

**Fig. 1:**
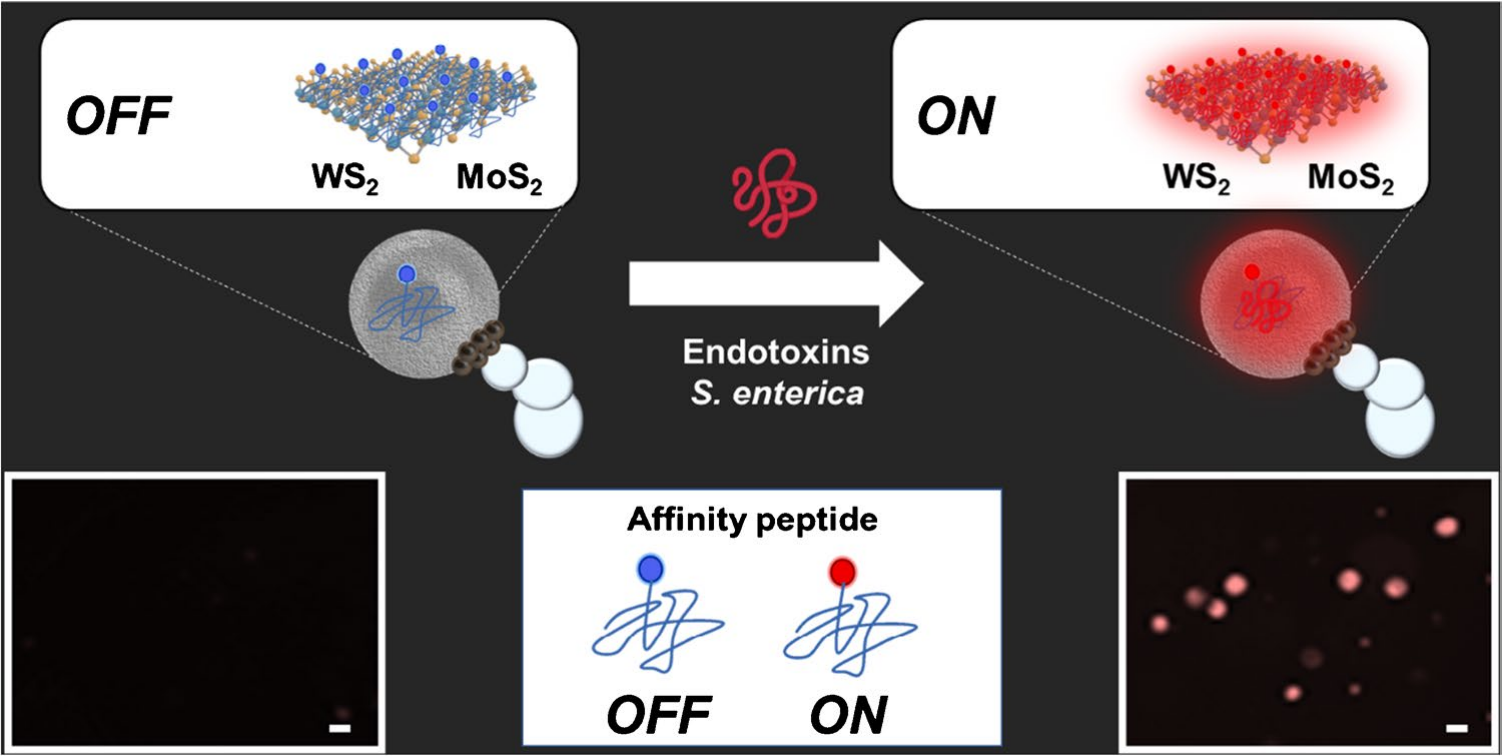
Salmonella enterica endotoxin detection strategy using affinity peptide-modified Janus micromotors. The lower part of the figure shows fluorescence microscopy images before (*OFF*) and after detection (*ON*). Conditions, 300 μg/ml endotoxin, 5% PEG, 7.5% H_2_O_2_. Scale bars, 10 μm

The detection strategy using Janus micromotors is shown in Fig. 1. The approach is based on an *OFF–ON* fluorescence optical detection setup. In the *OFF state*, the fluorescently labeled affinity peptide is bound to the 2D TMD nanomaterial, WS_2_ or MoS_2_, encapsulated in the micromotor, resulting in quenching of its native fluorescence (left image) [27]. In the presence of the specific endotoxin of *Salmonella enterica* serovar Typhimurium, the higher affinity of the peptide for the analyte causes it to detach from the TMD, bind to it, and produce a recovery of fluorescence inside the micromotor (right image). This increase in the fluorescent intensity of the micromotor is directly proportional to the concentration of the analyte in the solution and can be used for quantitative purposes.

Janus micromotors are synthesized using an emulsion self-assembly technique employing PCL as the main constituent polymer of the micromotor (for details on the synthesis, see the “Materials and Methods” section and Figure S1 in the supporting information). This technique allows the adequate encapsulation of different nanomaterials or precursors to achieve different propulsion mechanisms or applications to the system. It consists of the generation of an emulsion by mixing an aqueous solution that contains the TMDs (WS_2_ or MoS_2_) and a surfactant to decrease the interfacial tension in the emulsion, together with an organic solution that contains the polymer and nanoparticles responsible for propulsion (see step 1 in Figure S1). The mixture is vigorously stirred to generate microemulsions, which as the organic phase is allowed to evaporate solidifies the polymer, forming small PCL spheres with the nanoparticles accumulated asymmetrically in one area of ​​the sphere, generating the Janus character that will allow their mobility (see step 2 in Figure S1). Platinum nanoparticles are employed for the catalytic bubble propulsion by decomposition of H_2_O_2_ fuel, generating oxygen bubbles. Iron oxide nanoparticles are also incorporated in the micromotor to impart it with magnetic properties to facilitate washing steps and potential reusability.

**Fig. 2:**
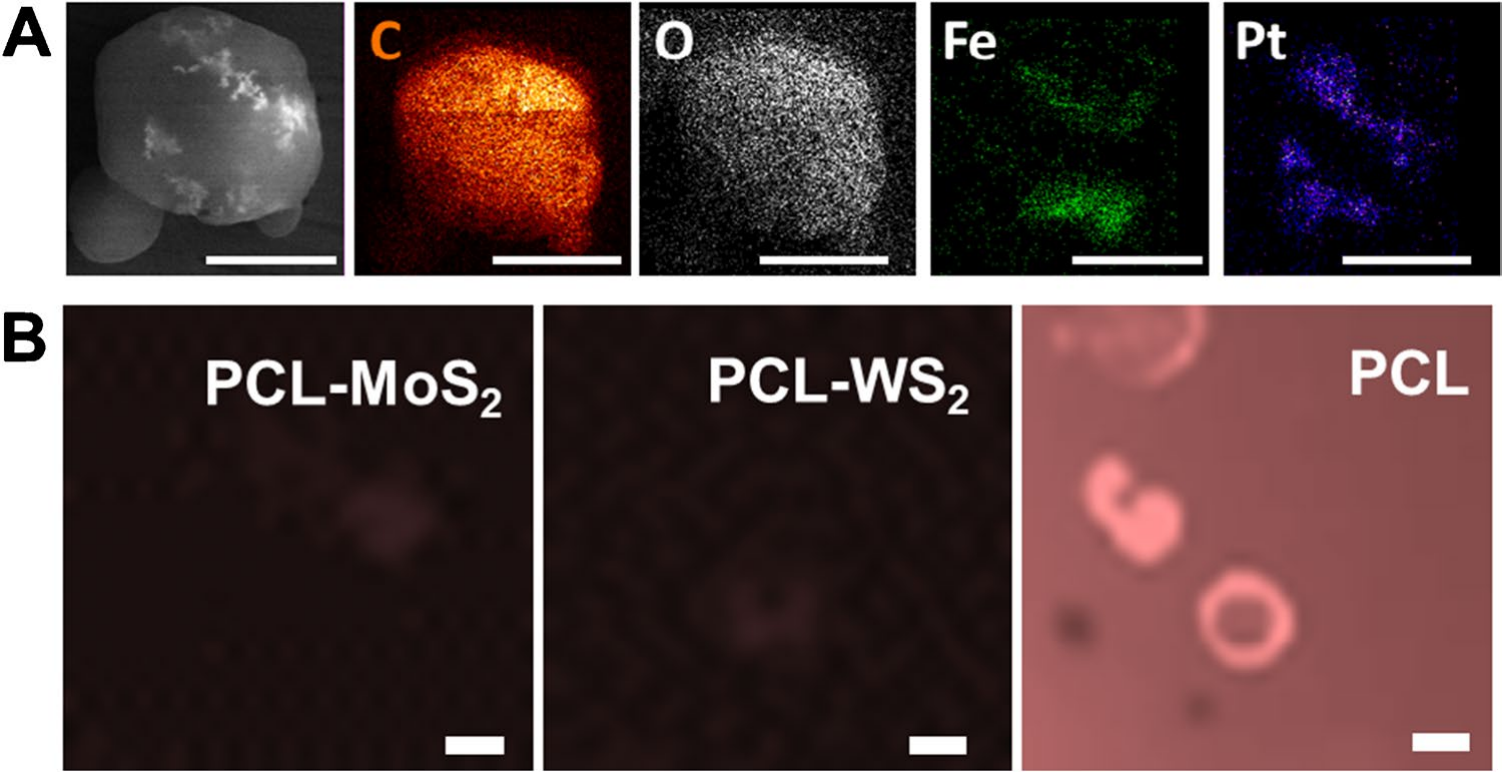
**A** SEM and EDX images showing the asymmetric distribution of the catalytic patch in the micromotors. **B** Microscopy images of the PCL micromotors and controls after incubation with the affinity peptide. Scale bars, 5 μm

Once the micromotors were synthesized, the morphology and correct encapsulation of the nanoparticles and 2D nanomaterials were evaluated by SEM and EDX mapping. As shown in the images and the table in Figure S2, the micromotors have a spherical structure with a mean diameter of less than 10 µm and an asymmetric region corresponding to the catalytic and magnetic nanoparticles, and they show a similar approximate speeds of 25.4 ± 6.9 µm/s and 28.6 ± 6.5 µm/s for the MoS_2_ and WS_2_ micromotors, respectively, when navigating in a solution containing 5% of PEG as the surfactant and hydrogen peroxide 7.5% as catalyst fuel. Note in Video S1 the importance of using a surfactant, reducing the surface tension of the fluid, and allowing the proper generation of gas bubbles produced by the decomposition of H_2_O_2_ into water and O_2_. Additionally, as can be seen in Fig. 2A, EDX studies of the micromotors were carried out, where the polymer body (C and O) of the micromotor and its asymmetric Fe and Pt patch are observed. Also, Figure S3 shows another micromotor where specific spots were selected for EDX mapping. We selected an area outside the asymmetric patch of the micromotor, where we detect Mo and W, respectively, corresponding to the 2D TMDs nanomaterials on the micromotors. Next, we select one spot on the asymmetric nanoparticles patch, detecting Fe and Pt, which are distributed on one side of the micromotor. Additionally, fluorescence microscopy studies were performed to assess modification with the affinity peptide. As can be seen in Figure S4, the micromotors encapsulating the 2D nanomaterials can absorb the affinity peptide on their surface in just 5 min of incubation, producing a decrease in the fluorescence of the surrounding solution (in blue). However, as can be seen in Fig. 2B in the case of unmodified PCL micromotors (without TMDs), the entire medium is fluorescence, indicating that there is no encapsulation of the peptide even within 1 h, compared with the low time require with the TMDs Janus micromotors. These data show successful modification with the TMDs and successful encapsulation of the peptide.

**Fig. 3:**
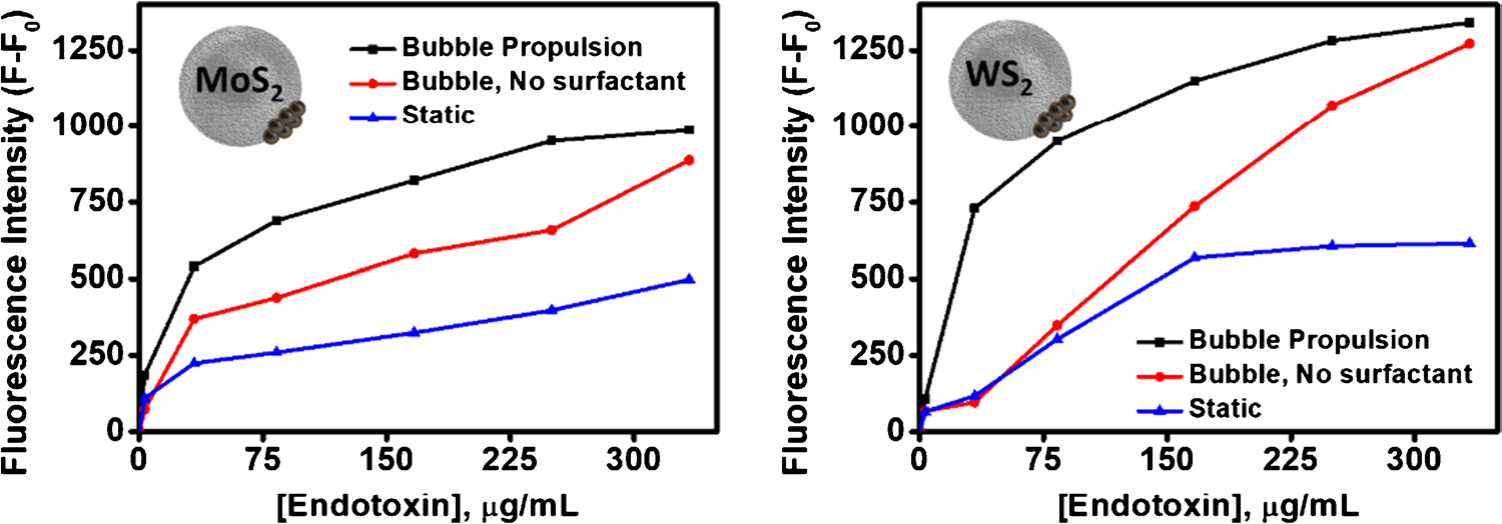
Fluorescence intensity of Janus micromotors based on TMDs nanomaterials and modified with the affinity peptide in the presence of *Salmonella enterica* serovar Typhimurium endotoxin under different conditions: bubble propulsion using surfactant (black), bubble propulsion without surfactant (red), and static (blue). Conditions: 5% PEG, 7.5% H_2_O_2_

Prior to study the analytical characteristics for MoS_2_ or WS_2_ Janus micromotors, the critical role of micromotor motion on induced fluid motion and its influence on micromotor fluorescence intensity was studied. As can be seen in Fig. 3, the fluorescence intensity of the micromotors increases with increasing endotoxin concentration (0–333 µg/mL) in just 5 min of analysis (black line). This increase is also observed using moving micromotors without adding a surfactant (PEG); however, the fluorescence intensity is lower since the absence of surfactant prevents the correct formation of oxygen bubbles and the efficient micromotor propulsion (red line; see Video S1). This reduces the mixing of fluids, resulting in a longer detection time and lower sensitivity. In the case of static micromotors (blue line), no increase in fluorescence intensity is observed given the absence of a propulsion mechanism. These results highlight the critical role of improved micromotor movement in improving the analytical performance of the detection strategy [28]. Please note here that the amount of PEG surfactant was evaluated at different concentration (1%, 3%, 5%, and 10%) using a fix concentration of 7.5% of H_2_O_2._ From 1 to 3% of PEG, no efficient bubble evolution was noted, which resulted in negligible micromotor motion. Efficient movement was noted using 5% and 10% PEG. In order to decrease the amount of reagents used, 5% of PEG was used. Following this principle, 7.5% H_2_O_2_ concentration was selected, which is a middle point amounting the best detection performance and reducing the use of peroxide, which can be incompatible with certain samples at high concentrations.

**Fig. 4:**
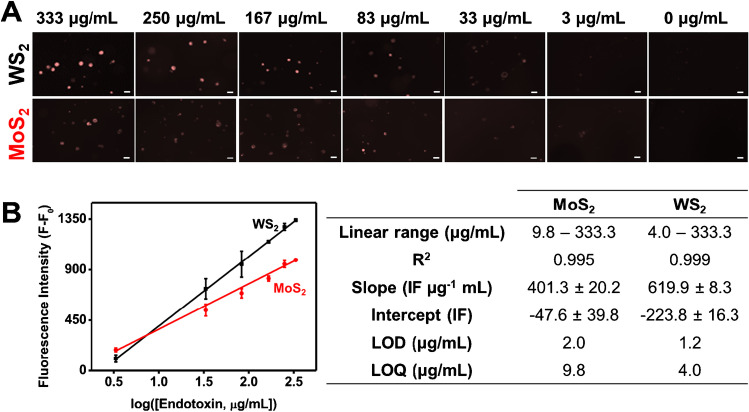
**A** Fluorescence microscopy images of affinity peptide-modified TMDs Janus micromotors in the presence of increasing concentrations of *Salmonella enterica* serovar Typhimurium endotoxin. **B** Corresponding calibration plots and table with analytical characteristics. Conditions, 5% PEG, 7.5% H_2_O_2_. Scale bars, 10 μm

Then, the calibration plots were obtained using different concentrations of *Salmonella enterica* serovar Typhimurium endotoxins. Figure 4A shows the fluorescence images after 5-min navigation of the affinity peptide-modified Janus micromotors in solutions with increasing endotoxin concentrations. The fluorescence intensity data were related to the analyte concentration to obtain the calibration line, which fits a logarithmic model (Fig. 4B) for both micromotors. Fluorescence analysis comes from the measurement of three independent drops (*n* = 3 drops/assay). For each drop, fluorescence measurement is taken from the average of the individually measured fluorescence in a representative number of micromotors (*n* = 50 motors approx./drop). Finally, the fluorescence of the assay is the average of the fluorescence obtained in each of the drops. Figure S5 shows WS_2_ and MoS_2_ micromotor photos taken from three independent drops using an endotoxin concentration of 83 μg/mL. As shown in Fig. 4B, the analytical performance of both WS_2_ and MoS_2_ micromotors was excellent, with good linear ranges and similar sensitivities for the analysis of this endotoxin. Indeed, an effect of the type of nanomaterial encapsulated in the micromotor is observed, and the sensitivity is slightly higher when WS_2_ is used, probably due to the fact that this nanomaterial has a higher roughness and encapsulates a greater amount of affinity peptide [24].

**Fig. 5:**
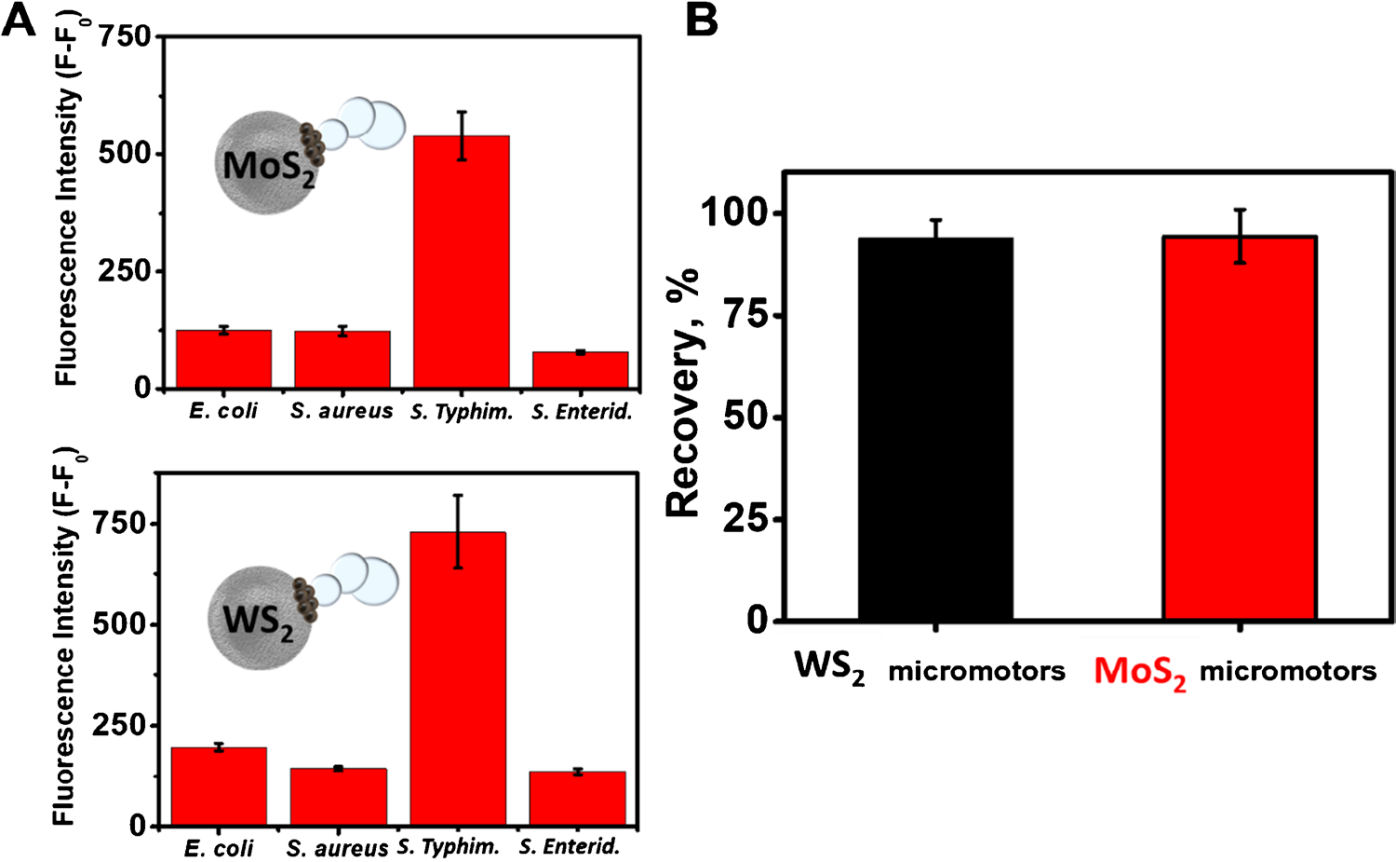
**A** Selectivity of the strategy in the presence of *Escherichia coli*, *Staphylococcus aureus*, and *Salmonella enterica* serovar Enteriditis endotoxins. **B** Recovery percentages were obtained with the Janus micromotor-based strategy in bacterial cultures fortified with 33 μg/mL of endotoxin (n = 3). Conditions, 5% PEG, 7.5% H_2_O_2_

Once the analytical characteristics were established, the selectivity of the method and the applicability in real samples were also evaluated. The evaluation of selectivity was carried out by studying the recovery of fluorescence in the presence of bacterial cultures of other Gram-negative bacteria and in relation to their structure, such as *Escherichia coli* and *Staphylococcus Aureus* and concerning the variations in the antigen O part of the endotoxin structure that allows discriminating between serovars, studying the specificity of 50 µg/mL *Salmonella enterica* serovar Typhimurium vs *Salmonella enterica* serovar Enteriditis. As shown in Fig. 5A, the strategy is highly selective as evidenced by the absence of fluorescence in the MoS_2_ and WS_2_ micromotors in contact with endotoxins from other bacteria and highly specific serotypes against *Salmonella enterica* serovar Enteriditis compared to endotoxins from *Salmonella enterica* serovar Typhimurium, thus, demonstrating the high selectivity of the strategy using specific affinity peptides for a bacterial type. For the recovery study in real samples, a culture medium was fortified with 33.3 µg/mL of *Salmonella enterica* serovar Typhimurium endotoxin, obtaining recovery percentages close to 100% in both cases (Fig. 5B). Due to the higher viscosity of the samples in the bacterial cultures and the presence of nutrients and salts, there was a drastic decrease in the speed of the micromotor from 25.4 ± 6.9 μm/s and 28.6 ± 6.5 μm/s to approximately 7.2 ± 1.3 μm/s and 12.1 ± 2.0 μm/s for the MoS_2_ and WS_2_ micromotors, respectively (see Video S2). However, this decrease in speed did not affect the practice application of micromotors, obtaining high recoveries in both cases.

Table 1 shows a comparison of developed methods for the determination of *Salmonella enterica* endotoxins. The limit of detection obtained in our work is slightly lower, but it allows to monitor *Salmonella* endotoxin contamination established in the legislation (275 μg/mL) [29]. Yet, the detection time required in our method (5 min) is lower than those reported for previous works (10–30 min). In addition, compared with similar based detection strategies using graphene oxide as a quencher, the analysis time and sample volume required in our method are lower than that obtained using fluorescently-labeled aptamers (30 min, 50 µL) [30] and bacteria-specific and selective than our previous work based on GQDs Janus micromotors [23]. Also, the developed method only requires 5 min of incubation with the affinity peptide; however, chip or microfluidic paper-based methods using antibodies require long periods (more than 1 h) for pretreatment, blocking, and incubation with the antibodies [31–33]. Furthermore, the use of affinity peptides avoids the use of labile and expensive antibodies with satisfactory results.

**Table 1:** Comparison of methods for *Salmonella enterica* endotoxins detection

Detection	Sensing probe	LOD	Linear range	Analysis time	Sample volume	Ref
Electrochemical (impedance)	Magnetosome-antibody complex	0.001 µg/mL	NR	30 min	10 µL	[34]
Electrochemical (impedance)	Magnetic nanoparticles, macrophage	0.15 µg/mL	1–50 µg/mL	3 h	NR	[35]
Fluorescent (*ON–OFF*)	GQDs Janus micromotors	0.07 ng/mL	0.2–3.5 ng/mL	15 min	4µL	[23]
Colorimetric	Antibodies-lateral-flow-assays	10 ng/mL	NR	15 min	In flow	[31]
White light reflectance spectroscopy	Antibody in chip coated LPS	4 ng/mL	10–10,000 ng/mL	15 min	In flow	[32]
Fluorescence polarization	Truncated aptamer-based GO	38.7 ng/mL	NR	30 min	50 µL	[30]
Electrochemical	Paper-supported 3D macrophage cell	0.0035 ng/mL	0.01–3 ng/mL	30 min	NR	[33]
Electrochemical (impedance)	Aptamer-conjugated gold nanoparticles	0.005 ng/mL	0.01–10.24 ng/mL	10 min	NR	[36]
Fluorescence (*OFF–ON*)	WS_2_ Janus micromotors	1.2 µg/mL	4–333.3 µg/mL	5 min	4 µL	This work
	MoS_2_ Janus micromotors	2.0 µg/mL	9.8–333.3 µg/mL			

*NR* Not reported.

## Conclusions

We have described an *OFF–ON* optical fluorescence mobile sensor based on catalytic Janus micromotor combining TMD nanomaterials for the entrapment of the recognition (bio)-receptor (fluorescently labeled affinity peptides) with high specificity for the detection of *Salmonella enterica* serovar Typhimurium endotoxins. Affinity peptides are bound to the 2D nanomaterial in the micromotor (*OFF* state) which recovers its fluorescence in the presence of the target toxin, increasing the fluorescence in the micromotor (*ON* state). The designed TMD Janus micromotor-based approaches showed high selectivity and sensitivity for *Salmonella enterica* serovar Typhimurium endotoxin analysis. The development of (bio)-sensing approaches using micromotor technology allows an improvement in the mixing of the fluid that lies in an increase in the speed of the analyte-receptor reaction, producing a rapid *on-the-fly* detection within 5 min in low volume of sample and obtaining quantitative recoveries in samples such as bacterial cultures. The strategy can be improved by using different receptors labeled with other fluorophores and specific against toxins from other pathogens for the development of a multiplex platform for its application in the detection of contaminants for the food safety, clinical, or environmental industry. In addition, these micromotor-based (bio)-sensing approaches can be combined with miniaturized technology, such as microplate readers or smartphones with optical detection for the development of fast, sensitive, and portable devices.

## Supplementary Information

Below is the link to the electronic supplementary material.

Supplementary file1 (MP4 1246 KB)

Supplementary file2 (MP4 5370 KB)

Supplementary file3 (DOCX 1634 KB)

## Data Availability

Not applicable.
