# Supplementary material for: Transition metal dichalcogenide-based Janus micromotors for on-the-fly Salmonella detection
Source: Mikrochim Acta. 2022 Apr 15;189(5):194. doi: 10.1007/s00604-022-05298-2 (PMC9010330; doi:10.1007/s00604-022-05298-2)
Supplement: Supplementary file 3 — Supplementary file3 (DOCX 1634 KB) [file 604_2022_5298_MOESM3_ESM.docx]

Electronic Supplementary Material

Transition metal dichalcogenides based Janus micromotors for on-the-fly *Salmonella* detection

Marta Pacheco,^1^ Beatriz Jurado-Sánchez,^1,2^* Alberto Escarpa,^1,2^*

^1^ Department of Analytical Chemistry, Physical Chemistry and Chemical Engineering, University of Alcala, Alcala de Henares E-28871, Madrid, Spain

^2^ Chemical Research Institute "Andrés M. del Río”, University of Alcala, Alcala de Henares E-28871, Madrid, Spain

***** Correspondence: beatriz.jurado@uah.es; alberto.escarpa@uah.es. Phone: +34 91 8854995

**Supporting Videos**

**Video S1.** Micromotors navigating in a hydrogen peroxide solution with and without surfactant (PEG).

**Video S2.** Micromotors swimming in culture media.

**Supporting Figures**

**
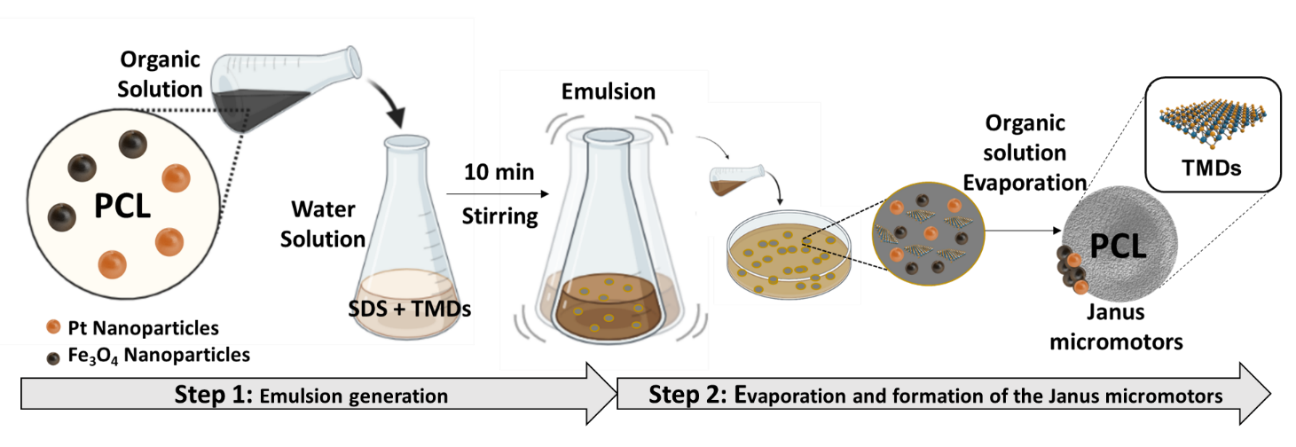
**

**Figure S1.** Synthesis of catalytic Janus micromotors encapsulating TMDs using the emulsion self-assembly technique.


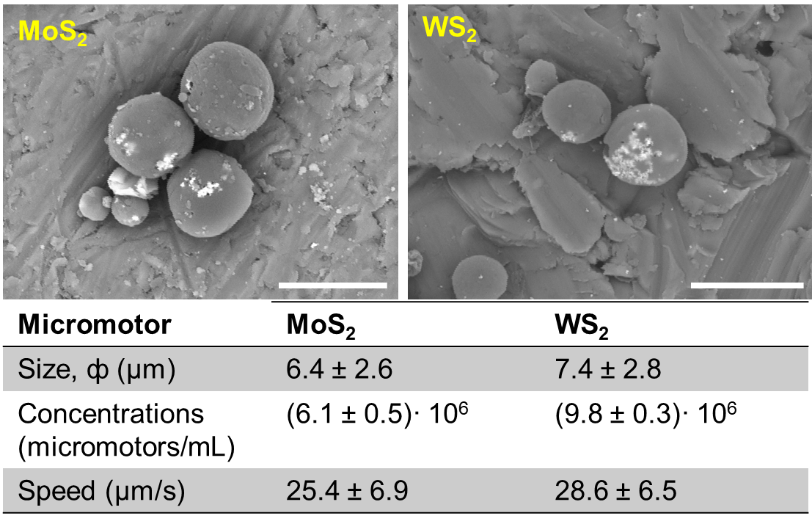


**Figure S2.** SEM images of the morphology of the TMDs Janus micromotors. The lower part shows a table with the average size of the micromotors, the number per batch and the speed of these in solutions containing 7.5% H_2_O_2_ and 5% polyethylene glycol. Scale bars, 10 μm.

**
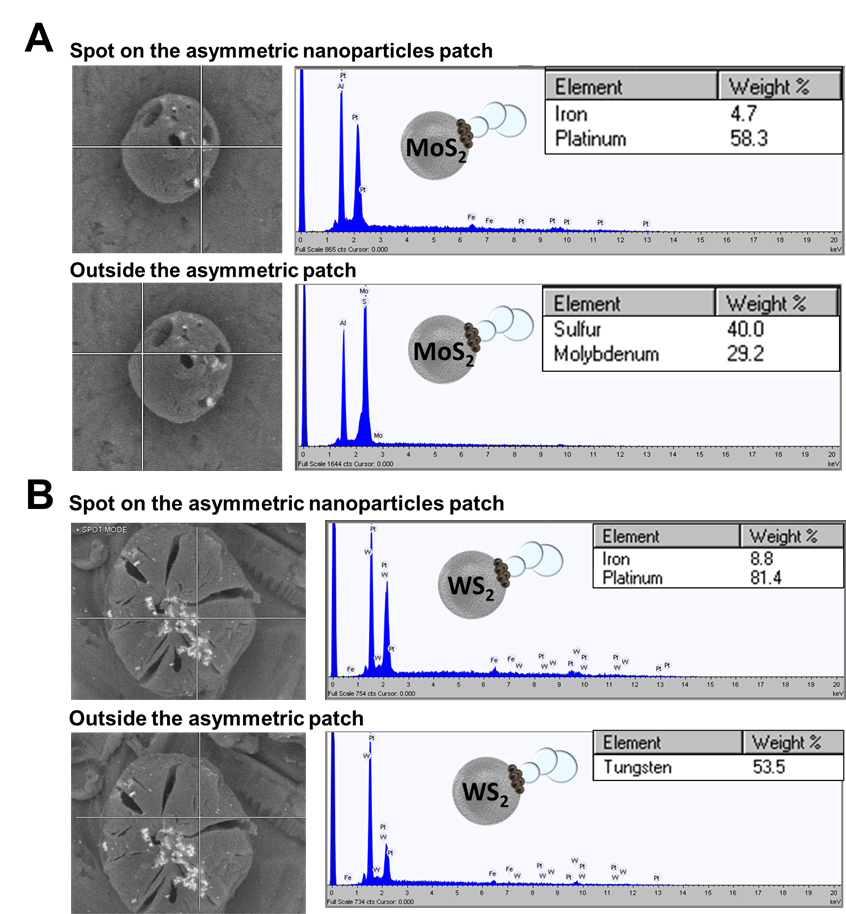
**

**Figure S3.** Spot SEM-EDX analysis in the asymmetric side of the nanoparticle patch and outside of the asymmetric patch of the Janus micromotors MoS_2_ (A) and WS_2_ (B).


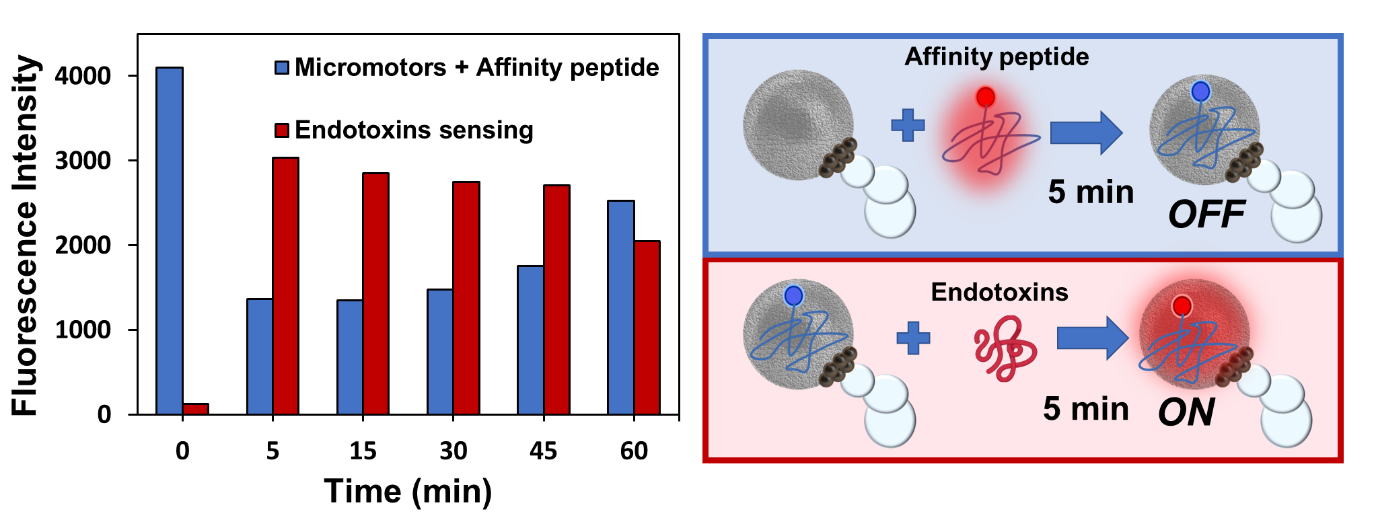


**Figure S4.** Optimization of the absorption time of the fluorescently labeled affinity peptide on the surface of the Janus micromotors with 2D TMDs nanomaterial, the graph shows the fluorescence intensity in the solution surrounding the micromotors (in blue); and time required to recover fluorescence in the micromotors after endotoxin detection (in red).


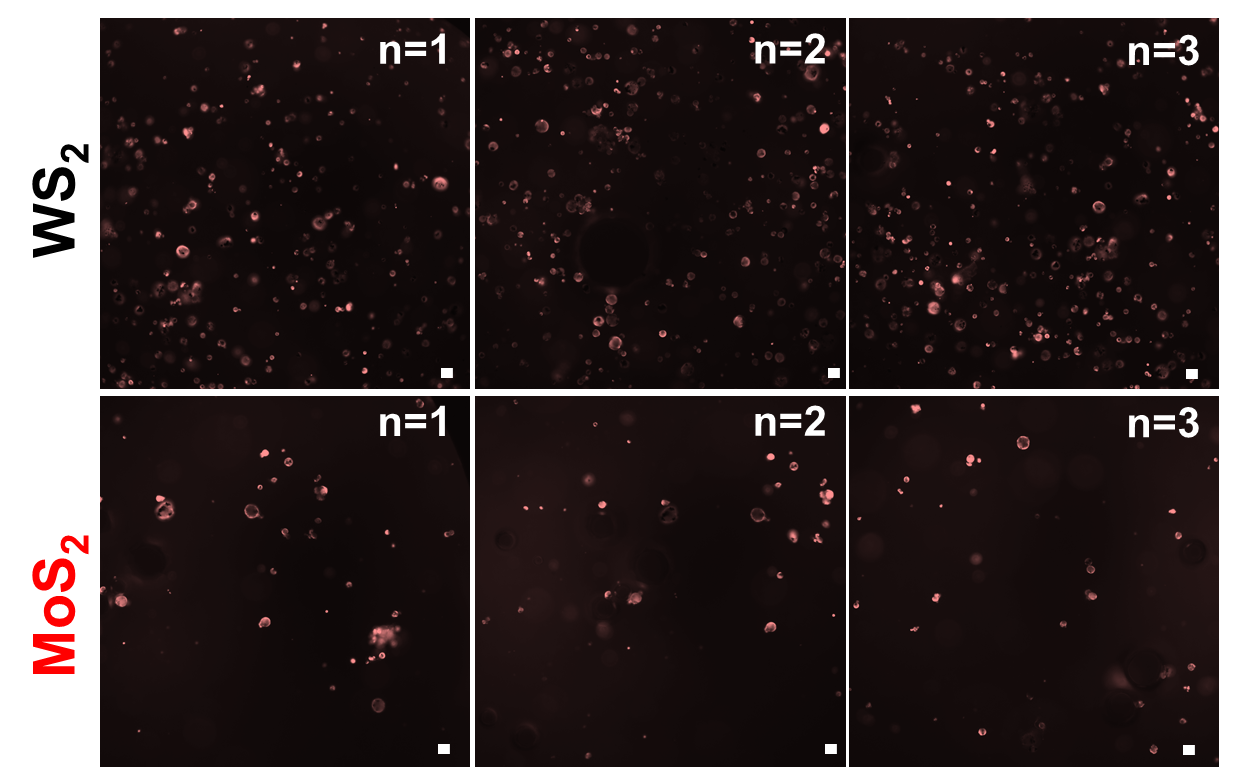


**Figure S5.** Time-lapse fluorescence images of the WS_2_ and MoS_2_ micromotors from three different drops after the detection of 83 μg/mL endotoxin. Scale bars, 10 μm.
